# Noninvasive Label-Free Detection of Cortisol and Lactate Using Graphene Embedded Screen-Printed Electrode

**DOI:** 10.1007/s40820-018-0193-5

**Published:** 2018-03-02

**Authors:** Satish K. Tuteja, Connor Ormsby, Suresh Neethirajan

**Affiliations:** BioNano Lab, School of Engineering, University of Guelph, Guelph, ON N1G 2W1 Canada

**Keywords:** Graphene, Immunosensor, Electrochemical, Screen-printed electrodes, Cortisol sensor

## Abstract

**Abstract:**

A sensitive and specific immunosensor for the detection of the hormones cortisol and lactate in human or animal biological fluids, such as sweat and saliva, was devised using the label-free electrochemical chronoamperometric technique. By using these fluids instead of blood, the biosensor becomes noninvasive and is less stressful to the end user, who may be a small child or a farm animal. Electroreduced graphene oxide (e-RGO) was used as a synergistic platform for signal amplification and template for bioconjugation for the sensing mechanism on a screen-printed electrode. The cortisol and lactate antibodies were bioconjugated to the e-RGO using covalent carbodiimide chemistry. Label-free electrochemical chronoamperometric detection was used to analyze the response to the desired biomolecules over the wide detection range. A detection limit of 0.1 ng mL^−1^ for cortisol and 0.1 mM for lactate was established and a correlation between concentration and current was observed. A portable, handheld potentiostat assembled with Bluetooth communication and battery operation enables the developed system for point-of-care applications. A sandwich-like structure containing the sensing mechanisms as a prototype was designed to secure the biosensor to skin and use capillary action to draw sweat or other fluids toward the sensing mechanism. Overall, the immunosensor shows remarkable specificity, sensitivity as well as the noninvasive and point-of-care capabilities and allows the biosensor to be used as a versatile sensing platform in both developed and developing countries.

**Graphical Abstract:**

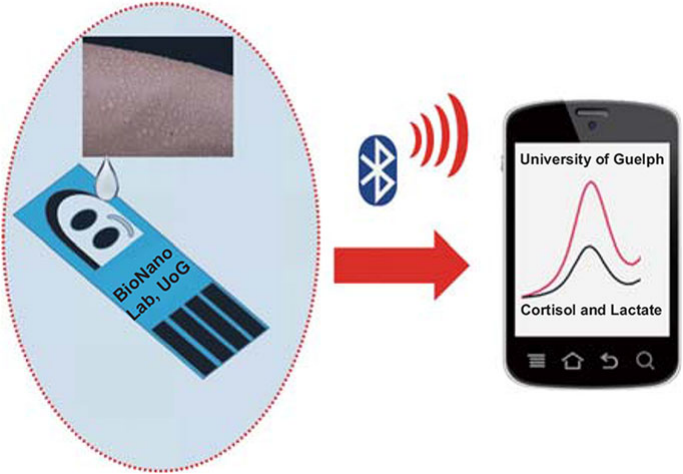

**Electronic supplementary material:**

The online version of this article (10.1007/s40820-018-0193-5) contains supplementary material, which is available to authorized users.

## Highlights

- The as-electrodeposited 2D nanosheets exhibit enhanced electroconductive nature.
- For the first time, a dual cortisol and lactate antibody-conjugated graphene biointerface has been developed. The developed assay is label free and applicable for the analysis of clinical samples.
- The results are well correlated and validated with existing commercial ELISA kit. The time of detection is very low at ≤1 min.

## Introduction

Cortisol is a glucocorticoid class hormone that is secreted by the zona fasciculata of the adrenal glands [1]. Its involvement in many physiological functions, such as amino acid and fat mobilization for metabolism, electrolyte balance, anti-inflammatory effects, and immune system suppression, makes it a useful biomarker for point-of-care (POC) diagnostics and health monitoring [2]. In addition to these functions, cortisol’s role in human cognitive behavior, such as working memory, sleep patterns, and mood, further demonstrates the complex functions of the hormone [1].

Lactate, a waste product of glycolysis, is produced by lactate dehydrogenase under anaerobic conditions [3]. Under normal conditions, the liver absorbs 70% of the lactate while skeletal and cardiac muscles remove the remainder [4]. But under abnormal conditions, such as liver necrosis, the liver fails to absorb the lactate, which can cause muscle fatigue and acidosis, leading to hypoxia and necrosis of other tissue types [5]. Factors affecting the liver’s ability to metabolize lactate are hepatic blood flow, oxygen supply, and chronic liver disease [5]. Increased lactate levels can also be attributed to cardiac arrest, sepsis, and intestinal infarcts [6].

Current techniques for measuring cortisol, such as enzyme-linked immunosorbent assay (ELISA), have many advantages including high sensitivity and specificity to the desired molecule. But the disadvantages that come with these methods can offset the benefits and detract from the monitoring of cortisol as a biomarker. These disadvantages include the necessity of shipping samples to a laboratory for analysis, the complex experimental procedures, and equipment needed to perform the testing [7]. Methods for the measurement of lactate in the blood possess the same disadvantages as measuring cortisol; taking blood samples, expensive and bulky laboratory equipment, and lengthy processing time make lactate monitoring difficult [8]. For cortisol and lactate to be monitored, a POC device is needed to allow for on-site testing.

With the current trend for smart, wearable technology being used to monitor the fitness level of its user by monitoring heart rate and steps taken, the market for these technologies has grown significantly. With a forecasted market size of US $5.8 billion in 2018, US $34 billion by 2020, and US $75 billion by 2022, the wearable technology trend has branched into other fields, including medical, communication, and lifestyle [9–11]. These technologies have also allowed for consistent monitoring of various health indicators, such as heart rate and electrical activity, blood glucose levels, and brain wave activity [12]. Many of these devices use Bluetooth technology to transmit the collected data to a smartphone with a specifically designed mobile application to receive the data, interpret the signal, and display a final, meaningful value to the user [8, 13, 14]. Another benefit of some of these POC devices is that it is not necessary to pierce the skin to obtain a sample of blood or tissue [8]. By using body fluids like sweat and saliva, rather than blood, noninvasive methods can be used to determine composition changes in these fluids. Correlations can then be made between the composition and internal body conditions, as an early warning sign of a disease or physiological stress in the body.

The development of a noninvasive, POC method to measure cortisol and lactate in body fluids can permit the monitoring of these biomarkers as an early warning sign of disease and stress. Previously, biosensors have been developed to detect each of these molecules separately. Sun et al. showed the capability of a cortisol biosensor using reduced graphene oxide and gold nanoparticles as the base of the bioassay [7]. An excellent detection limit and a wide range of detection of 0.1 to 1000 ng mL^−1^ was obtained by the biosensor developed [7]. Khan et al. and Kim et al. [15, 16] were also able to fabricate very sensitive biosensors with ranges of 3 pg mL^−1^–10 μg mL^−1^ and 10 pM–100 nM, respectively, using similar methods. Lactate biosensors developed by Azzouzi et al., using gold nanoparticles and lactate dehydrogenase, show a range of detection of 10 μM–5 mM with a detection limit 0.13 μM [17]. Labroo and Cui [18] developed a biosensor to detect 4 analytes: glucose, lactate, xanthine, and cholesterol, with a detection range of 0.3–15 μM.

The biosensor can be used in the medical, agricultural, and sports medicine industries. Monitoring cortisol and lactate in sports medicine can help to maximize training efficiency for professional athletes, so as not to over-train during workouts, or to monitor player health during a game or competition. This device can be used by farmers of all scales to monitor the health of their herd. By using other body fluids, such as saliva or lacrimal fluid, cortisol levels can be measured and connections can be made to the animal’s health and stress level. This can allow for better herd management as well as more effective isolation if an infectious disease breakout were to occur. Monitoring these biomarkers in humans can also show signs of disease and distress that can be used as an early warning sign of disease.

The recent surge in biosensors has been paralleled by the surge in the use of graphene in these biosensors and other applications. Graphene is a two-dimensional (2D) layer of carbon atoms, a single atom thick, bonded into a honeycomb lattice [19]. There are several properties that make this material ideal for use in sensors [20]. The first property is graphene’s ability to attract a wide range of aromatic molecules, due to the *π*–*π* interactions or electrostatic interaction [21]. Also, the copious amount of oxygen-containing functional groups present in graphene oxide (GO) can be manipulated from a variety of applications, such as medical imaging and pharmaceutical delivery. In optical biosensors, GO can fluoresce over a very large range of wavelengths, including near-infrared and ultraviolet [20]. GO can also quench the fluorescence of dyes [22]. Other properties, such as its electrical conductivity (1738 Siemens m^−1^), mechanical strength (1100 Gpa), and thermal conductivity (5000 W m^−1^ K^−1^), make GO an ideal material for use in sensors and biotechnology [23, 24].

Several objectives were set for the completion of this study. The first objective was to develop and characterize dual bioassays for the detection of cortisol and lactate, using antibodies, due to their interactions with the specific antigen molecules, to monitor them. Nanomaterials will be used in this step to facilitate the electrochemical nature of the biosensors. The next objective was to optimize the fabrication of the bioassays to determine a relationship between concentration and current. This was done by testing a range of concentrations of each of the analytes, determining a lower detection limit, and quantifying the specificity of the bioassays against other molecules. The final, ongoing objective is to develop the prototype as the integration of Bluetooth communication between a handheld potentiostat and a mobile app to transform the system into a POC monitoring device that does not rely on bulky laboratory equipment and complex procedures.

**Fig. 1:**
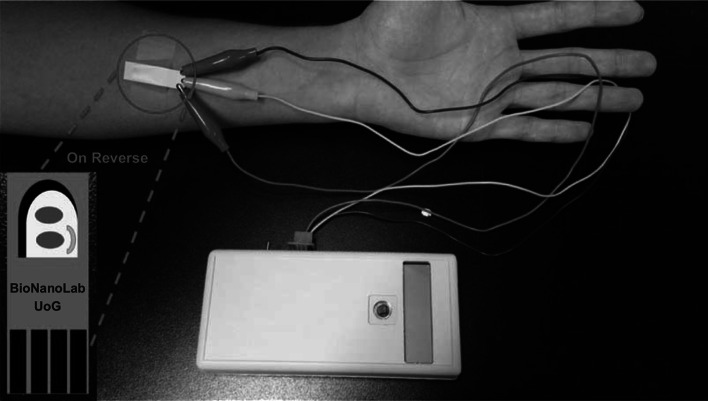
Schematic representation of a portable, handheld potentiostat assembled with Bluetooth communication and battery operation to allow for point-of-care applications. The prototype electrode is attached to patient’s arm and connected to handheld potentiostat to run test, and data will be sent to the mobile app to be displayed to the user

In this report, we present a noninvasive, POC method to measure both cortisol and lactate in sweat and other body fluids, using antibodies bioconjugated to reduced GO, using covalent carbodiimide chemistry on a screen-printed electrode. Also, to develop the prototype, a handheld potentiostat with Bluetooth capabilities and an Android OS-based mobile application were integrated to communicate the information to the user. A portable, handheld potentiostat assembled with Bluetooth communication and battery operation to allow for POC applications is presented in Fig. 1.

## Experimental Section

### Reagents

Graphene oxide, BSA (bovine serum albumin), N-hydroxysuccinimide (NHS), N-(3-dimethylaminopropyl)-N’-ethylcarbodiimide hydrochloride (EDC), cortisol solution, uric acid (UA), D(+) glucose (Glu), L-(+)-lactic acid solution (LA), L-ascorbic acid (AA), potassium hexacyanoferrite (K_3_[Fe(CN)_6_]), potassium hexacyanoferrate (K_4_[Fe(CN)_6_]), and phosphate-buffer saline (PBS) were purchased from Sigma-Aldrich (Oakville, ON). Anti-cortisol antibody [CORT-2] and anti-lactate dehydrogenase antibodies were purchased from Abcam (Cambridge, MA). Artificial sweat and saliva were purchased from Pickering Laboratories (Mountain View, CA). The deionized water of Milli-Q water (18.2 MΩ) was used in all experiments. The dual working carbon screen-printed electrodes (SPEs) used in this study were made by DropSens (model C1110, Spain). The working area (4 mm diameter) of these SPEs consisted of carbon-paste material, whereas carbon and Ag/AgCl were used to form the counter and reference electrodes, respectively.

### Characterization and Sample Preparation

The as-synthesized electroreduced graphene oxide (e-RGO) and e-RGO-modified SPE were characterized using imaging and spectroscopy methods to study the physical structure and composition of the materials. A transmission electron microscope (TEM-FEI Tecnai g2f20, 200 keV) and a scanning electron microscope (SEM-FEI Inspect S50, 15 V) were used to analyze the physical structures of the nanomaterials deposited. Atomic force microscopic (AFM) investigations were carried out in the non-contact mode using an AGILENT AFM/SPM 5500. Raman spectra were measured using a Renishaw spectrometer (514 nm excitation wavelength, 1.58 eV). For the Fourier transform infrared spectroscopy (FTIR) analysis, the Bruker Vertex 70-I (diamond ATR) was used to collect the data. The resolution was 4 cm^−1^ and 128 scans were performed to measure samples as well as the background. An energy-dispersive spectrometer (EDS) (Oxford Instruments X-Max 20) was used to estimate the chemical composition of the GO and e-RGO. The surface composition of the e-RGO-immobilized SPEs was studied by X-ray photoelectron spectroscopic (XPS) analysis using an Omicron XPS spectrometer (operating with an acceleration voltage of 12 kV and a power of 300 W). Measurement of the electrochemical parameters and the subsequent analysis were performed using a µStat400 Biopotentiostat with a 4-electrode connector from DropSens, Spain. Cyclic voltammograms (CVs) were recorded in the − 1 to + 1 V potential range at a scan rate of 50 mV s^−1^. Chronoamperometric detection (AD) responses were studied at an applied potential of 0.2 V. Origin Pro 16 (Origin Lab Corporation, MA, USA) was used for the preparation of graphs. A redox probe solution of 10 mM [Fe(CN)_6_]^3−^ + 10 mM [Fe(CN)_6_]^4−^ was prepared in 100 mM PBS. To prepare the samples for XPS, SEM, EDS, and FTIR, the GO and e-RGO were drop-cast onto silicon wafers and left to dry. For the SEM, EDS, and FTIR samples, once dried, another layer of GO and e-RGO was added to increase the thickness of the films produced. The samples for XPS were made on small silicon wafer chips (0.3 × 0.3 cm^2^) while the SEM, EDS, and FTIR samples were on larger chips (2 × 1 cm^2^). The samples for TEM were prepared with diluted GO and e-RGO on copper mesh grids.

### Graphene Embedded Electrode Fabrication and Characterization

Typically, an aqueous dispersion (1 mg mL^−1^) of 2D GO nanostructures was used for the electrodeposition over the working surface electrode of SPE and the same dispersion was utilized for further analytical characterization. The physical exfoliation of GO sheets was done by ultrasonication process to reduce the van der Waals forces present between the 2D layers of GO sheets. Afterward, 5 µL of exfoliated nanosheets was spread over the working area of SPEs and placed into a laboratory oven (Quincy Lab Model 10GC) at 110 °C for 1 h. After being left to cool for several minutes, the electroreduction was done by employing linear sweep voltammetry (LSV) electrochemical technique and CV. The three electrochemical reduction scans were given in 100 mM PBS buffer at scan rate of 0.1 V s^−1^ and step potential of 0.001 V in the range of 0 to − 1.4 V. The stability and successful deposition of electroreduced e-RGO over the SPE was achieved by running 25 cycles of CV (recorded in the − 1 to + 1 V potential range at a scan rate of 50 mV s^−1^). Further, the e-RGO was comprehensively analyzed by the spectroscopic and microscopic techniques.

### Antibodies Conjugation Over e-RGO and Biointerface Development

The successful attachment of the cortisol and lactate antibodies on the e-RGO SPEs was carried out via EDC-NHS carbodiimide covalent chemistry using EDS-NHS linkers. 10 μL of 10 µg mL^−1^ anti-cortisol/anti-lactate antibody (stock concentration 1 mg mL^−1^) solutions was spread over the e-RGO-modified SPEs and incubated for 2 h at 25 °C to form Ab@e-RGO. The intact carboxyl group presented on e-RGO facilitates the antibody covalent conjugation. The electrode surface and unbound antibodies were washed with 10 mM PBS solution. The non-specific protein interactions of modified SPE were minimized by incubating with blocking solution for 30 min. The blocking solution contained BSA solution (2 μL, 0.25% w/v) and finally SPE working surface washed with buffer solution. The antibody-modified SPE was then stored at 4 °C when not in use. The successful antibody conjugation on the e-RGO surface was characterized by spectroscopic and electrochemical techniques.

### Label-Free Electrochemical Quantification of Cortisol and Lactate Antigen

The developed Ab@e-RGO immunosensor was tested for its electrochemical response with respect to varying cortisol concentrations, i.e., 0.1–200 ng mL^−1^, and varying lactate concentrations, i.e., 0.5–25 mM. The antigen solutions were prepared in PBS (pH 7.4). Two additional sets of these concentrations were made using artificial sweat and artificial saliva. All samples were stored at 4 °C for later tests. A small volume (5 μL) of the antigen solution was exposed to the working electrode area of the immunosensor. It was then left to incubate for 2 min at room temperature to ensure the antibody–antigen binding. Then the electrode was washed by gentle immersion in the PBS buffer before analysis of its electrochemical properties with respect to the introduction of a redox probe electrolyte (50 μL of 10 mM [Fe(CN)_6_]^3−^ + 10 mM [Fe(CN)_6_]^4−^ was prepared in 100 mM PBS) [23]. The electrochemical chronoamperometric measurements were recorded to monitor the change in the current response of the prepared electrodes. Baselines were established by analyzing a blank control sample using PBS buffer only. Each of the concentration sets was tested on three different electrodes. The detection limit was determined according to the 3 s m^−1^ criteria, where s represents the standard deviation of the blank and m denotes the slope of the calibration plot.

### Interference Study

Due to the high number of components in body fluids such as sweat and saliva, an interference study was performed to determine the specificity of the bioassays to their respective biomarkers. The cross-reactivity specificity of the developed immunosensor electrodes was evaluated by investigating their response against non-specific interferents, namely AA, UA, and Glu. The selectivity study was done using separate sample individually of each interferent. Lactate and cortisol were also included to show that there is no cross-sensitivity between the two bioassays. The 1.2 mM fixed concentration of interferents was used for these tests. 10 μL of the interferents was pipetted onto the electrode surface. After 2-min incubation, an AD response of the immunosensor surface was recorded after incubating the non-specific analytes at a voltage of 0.2 V.

## Results and Discussion

**Fig. 2:**
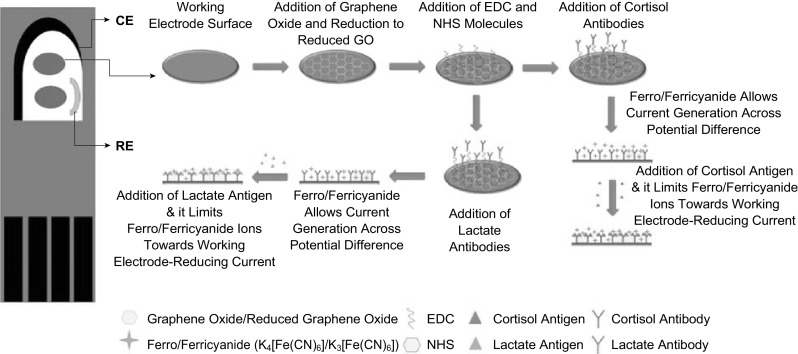
Schematic illustration of carbon screen-printed electrode: *CE* counter electrode, *RE* reference electrode. Working electrode surface is with sequence of surface modifications for bioassay fabrication. The e-RGO (electroreduced graphene oxide)-modified SPEs were then functionalized with cortisol and lactate antibodies to develop a biointerface for label-free electrochemical immunosensing

**Fig. 3:**
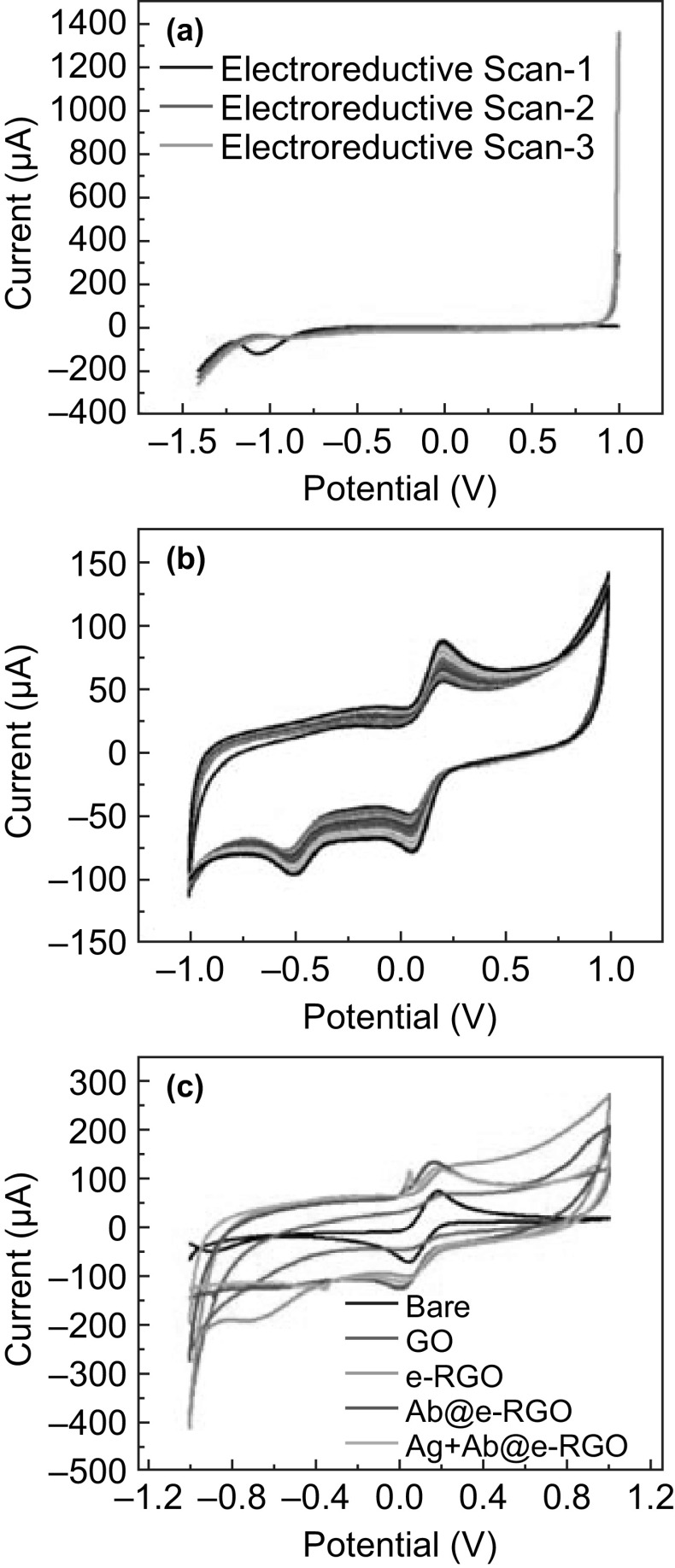
**a** LSV-based characterization of electroreduction steps of GO to e-RGO. **b** Electrodeposition of graphene by CV. **c** The CV curves at different stages of SPE modification

The step-by-step assembly of the e-RGO-based antibody immunosensor has been depicted in Fig. 2. The e-RGO on SPE was synthesized by the electrochemical reduction technique after successive reductive scans of GO in PBS. The electrochemical LSV reduction technique (potential reductive scans from 0 to − 1.4 V) is employed to reduce the hydroxyl, epoxy, and other oxygen-containing functional groups present in the parent structure of GO and helps to convert GO to e-RGO. As illustrated in Fig. 3a, the LSV peak starts to diminish after each reductive scan, leading to the e-RGO free from –OH and epoxy groups. The complete electrodeposition of e-RGO nanosheets was further achieved by employing 25 cycles of CV (Fig. 3b). The CV curves point toward the stable and reproducible electrodeposition of graphene over the working area of the SPEs without any divergence in the redox peaks. The *π*–*π* interactions between the graphitic electrode surface and hexagonal rings of graphene are strong enough to support the stability of electrodes, which provide additional force for the strong interaction of graphene layers with the carbon-based SPE. The CV response was checked at each step of immunosensor modification, and the CV characteristics illustrated in Fig. 3c are for the redox electrochemical reaction of ferro/ferri. The CV of e-RGO curves indicating a larger electroactive area and showed a great escalation in the peak current as compared to the bare GO-modified SPE. The modification of the SPE electrodes with graphene reveals the electroconducting and electroactive nature. The effective surface area of electrodes at different modification steps was calculated by Eq. 1 as described in Ref. [23]:

1 $$ {\text{ip}} = 2.69 \times 10^{5} n^{3/2} AD^{1/2} Cv^{1/2} n $$

where ip is peak current, *A* is electrode surface area, *n* is number of electrons transferred, *D* is diffusion coefficient, *C* is concentration of redox media, and *v* is scan rate. The estimated surface areas for the bare and e-RGO immobilized were 0.026 and 0.044 cm^2^, respectively. The step-by-step modification of SPE is also calculated by electrochemical impedance spectroscopy (EIS) and shown in Fig. S1.

**Fig. 4:**
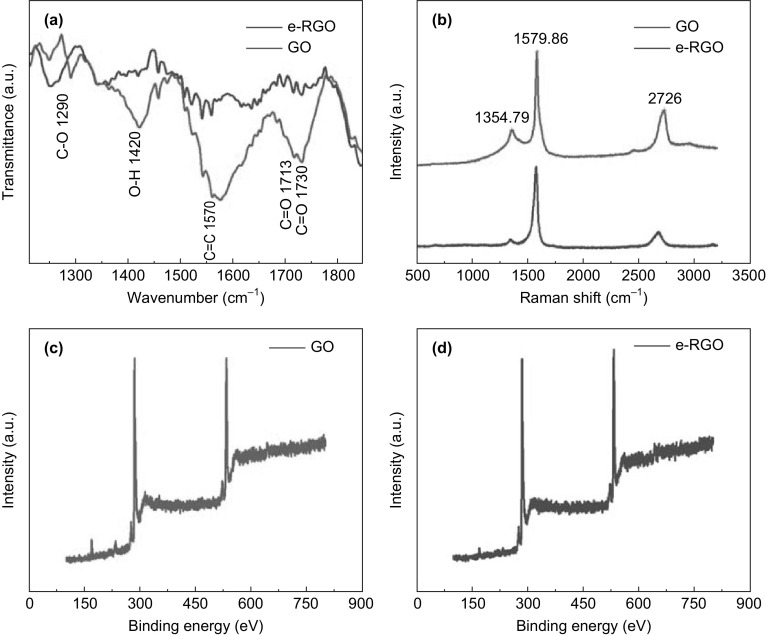
**a** Functionalization study of GO and e-RGO using FTIR analysis. **b** Raman spectra of GO and e-RGO. **c** XPS analysis of GO and e-RGO

The spectroscopic and structural characterization of features of the e-RGO-modified SPEs was investigated using FTIR, Raman spectroscopy, XPS, and EDS. The FTIR spectroscopy showed (Fig. 4a) the reduction in several peaks corresponding to different functional groups in the GO. Some of the peaks that were noticeably reduced were at the wavenumbers ~ 1713 and ~ 1730 cm^−1^ corresponding to C=O, ~ 1570 cm^−1^ corresponding to C=C, 1420 cm^−1^ corresponding to –OH functional group, and ~ 1290 cm^−1^ corresponding to C–O. There was also a large reduction around the wavenumber centered at ~ 3300 cm^−1^ which also corresponds to the -OH functional groups. The carboxyl group present in the structure of e-RGO gives stretching vibrations around ~ 1700 cm^−1^, and the –COOH group is responsible for the antibody conjugation [25–27].

**Fig. 5:**
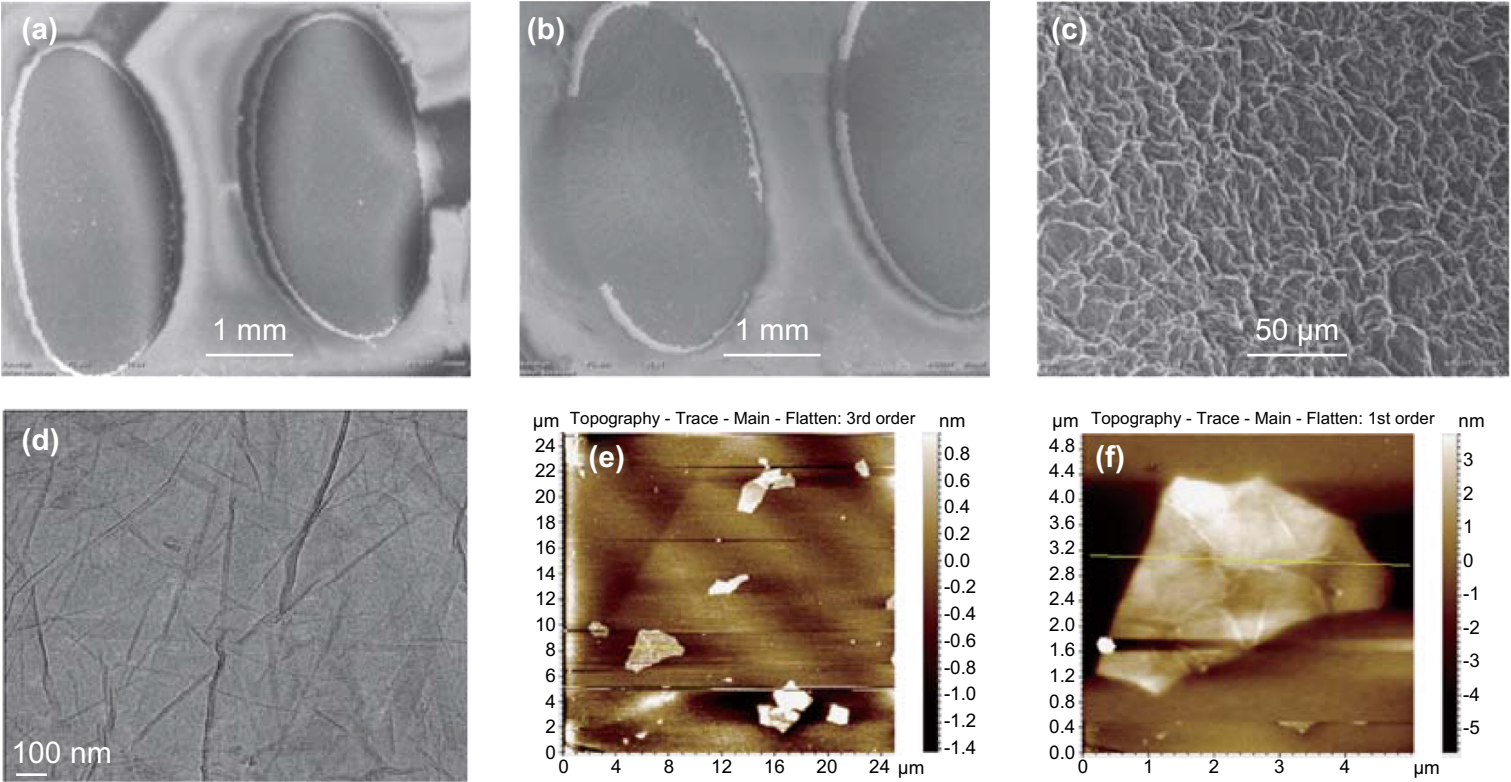
**a** SEM analysis of dual SPE before electrodeposition. **b** SEM analysis of SPE after electroreduction. **c** High magnification SEM images of electrodeposited e-RGO over the dual SPE. **d** TEM image of e-RGO. **e**, **f** AFM image of e-RGO

The type, intensity of the structural defects, and thickness of the electrodeposited e-RGO layers were studied by collecting the Raman spectrum (as illustrated in Fig. 4b). The spectra clearly show three characteristic bands of graphene nanostructure, a crystalline *G* band around ~ 1580 cm^−1^ attributed to vibrations of *sp*^2^ bonding, whereas the disordered *D* band around ~ 1355 cm^−1^ is corresponding to the scattering caused by the defects produced in the *sp*^2^ hybridized two-dimensional hexagonal lattice of graphene [28]. The third 2D band with the shape and width of 2D band number of graphene layers can be estimated. In spectra, e-RGO showed blue shift indicating the lesser number of graphene thickness due to the electroreduction as compared to GO. The *I*_D_/*I*_G_ ratio of e-RGO is 0.3 and its low enough to verify a crystalline, defects free in the structure of the e-RGO as compared to GO with *I*_D_/*I*_G_ ratio of 0.78 [29, 30]. The detailed XPS analysis of GO and e-RGO immobilized on the SPE are presented in Fig. 4c, d. Peaks around 285 eV are associated with the oxygenated functional groups, carbonyl, carboxyl and hydroxide [31, 32]. The peaks originating around 530 eV are assigned to C=O, C–OH, and O=C–OH. Further, these results obtained from XPS study are confirmed by EDS analysis. The sheet-like morphological appearance of the e-RGO was assessed by SEM and TEM imaging. The SEM investigation illustrated the micrograph images of the bare dual working SPE before electrodeposition (Fig. 5a) and e-RGO-modified SPE (Fig. 5b). The successful electrodeposition of graphene over the working area of the SPE is clearly depicted in the SEM analysis (shown in Fig. 5c) and electrodeposition over the dual SPEs was further investigated by using AFM and TEM analysis. For TEM analysis, the deposited material (e-RGO) was carefully separated out from one of the test electrodes. The TEM micrograph of e-RGO is given in Fig. 5d and it clearly indicates the successful electrodeposition. The corresponding EDS analysis (Fig. S2) highlights composition and makeup of GO and e-RGO materials. In the samples of GO, the weight percentages of carbon and oxygen were 56.9 and 43.1%, respectively. In the samples of e-RGO, the weight percent of carbon increased, while that of oxygen decreased, to 67.1 and 32.9%, respectively. AFM micrographs and the corresponding height profiling analysis manifest ~ 2 nm in height corresponding to 3–4 graphene layers (as shown in Fig. 5e, f), and the corresponding line mapping is shown in Fig. S3.

**Fig. 6:**
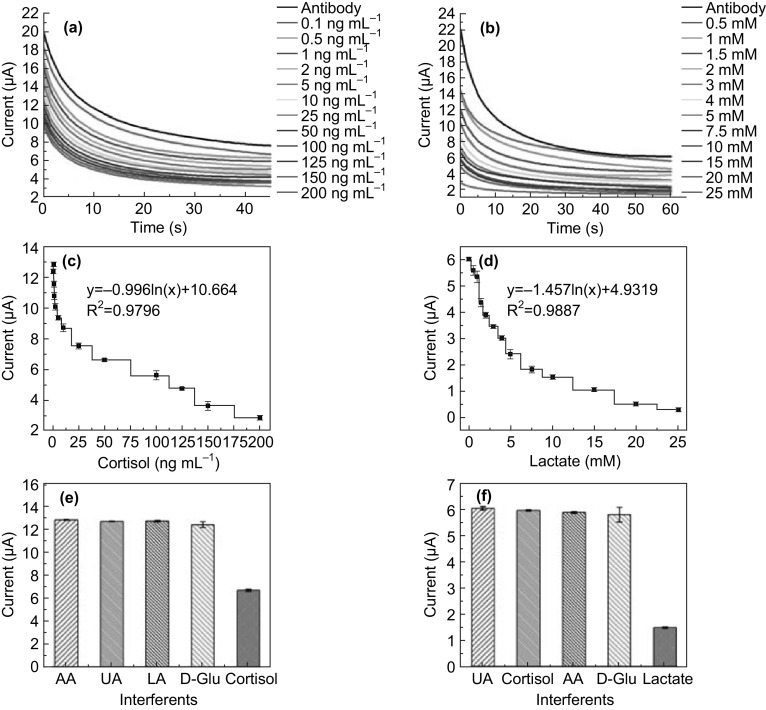
Chronoamperometric curve recorded from Ab@e-RGO SPE vs. different concentrations of **a** antigen cortisol, **b** antigen lactate. **c** Calibration curve from anti-cortisol Ab@e-RGO SPE against various concentrations of antigen cortisol. **d** Calibration curve from anti-lactate Ab@e-RGO SPE against various concentrations of antigen lactate. **e, f** Investigations of sensor response toward cortisol and lactate, respectively, in addition to other non-specific proteins, to assess the device’s specificity (concentration was fixed at 1 mM)

Herein, the applicability of the antibodies-modified e-RGO surface (Ab@e-RGO) in dual working electrode configuration has been employed for cortisol and lactate monitoring, using the chronoamperometric technique. Figure 6a, b shows AD recorded with the successive addition of aliquots of different concentrations of target cortisol and lactate antigen, respectively, over a wide analyte concentration range in a PBS buffer of pH 7.4, and Fig. 6c, d shows the respective calibration curves. In AD, the current intensity decreased gradually with the increase in the concentration of antigen. The validated wide range of analysis for cortisol was from 0.1 to 200 ng (*R*^2^ ~ 0.98, with a LOD ~ 0.1 ng) and lactate from 0.5 to 25 mM (*R*^2^ ~ 0.99, with a LOD ~ 0.110 mM) and able to detect the analytes over a very wide range. As the concentration of antigen increases, the current of the electrochemical response decreases, which is attributed to the insulating effect of the protein structure present in the antigen solution and shows the non-conducting behavior of the biomolecules. The antigen protein (cortisol and lactate) layers create a barrier for the diffusion and flow of Fe ions toward the working electrode of the SPE, which eventually decreases the peak current of AD attributed to the reduction in Fe^3+^ to Fe^2+^. As the protein concentration (cortisol and lactate) increases, it creates insulating protein layer on the electrode surface, which blocks the diffusion of the redox metal ion moieties. This passivation is due to the presence of dielectric insulating layers formed by the cortisol and lactate antibody–antigen complex. The antibody- and antigen-binding event generates electrochemical signals which are amplified by the 2D graphene structure immobilized on the working area of SPE. The interaction of biomolecules with graphene sheets introduces or withdraws the charge and influences the electrical properties of 2D graphene sheets. The adsorption of proteins such as antibodies and antigens over the surface of graphene sheets acts as dopants and perturbs the electronic properties of graphene, and this change may act as a mechanism for the detection of a biological event.

The Ab@e-RGO SPEs was also tested with spiked samples of cortisol and lactate in artificial sweat and saliva, and the observed current values from AD were correlated with the standard data and equations as shown in the calibration plots (Fig. 6c, d). The detailed results of the electrochemical analysis of cortisol and lactate by AD are given in Fig. S4, which indicates that Ab@e-RGO biointerface is useful for admirable analyzing the cortisol and lactate concentrations in spiked saliva and sweat samples. The validation of practical usability of the Ab@e-RGO/SPE for cortisol and lactate was then demonstrated by comparing the results obtained from commercial colorimetric kits for cortisol and lactate. The colorimetric assay was performed on the standard as well as on the spiked sweat and saliva samples. Standard graphs of absorbance vs. concentration of cortisol and lactate were plotted as shown in Fig. S5. The results obtained from AD techniques are consistent with the commercial kits in terms of sensitivity and showed the better limit of detection as compared to the commercial colorimetric kit. The AD method consumes a greatly reduced response time (less than ≤ 1 min) in comparison with the commercial colorimetric kit (≥ 6 h). The specificity of the developed immunosensor was then evaluated by conducting AD studies after incubating the immunosensor surface with some close structurally biological components as non-specific interferents (concentration was fixed at 1.2 mM), such as Glu, AA, and UA. As the results show (represented in Fig. 6e, f), the current generated across the potential showed a significant decrease in the respective biomarker only for each bioassay. The interferents used (d-glucose, ascorbic acid, and uric acid) showed no decrease current over the trails. It was also shown that the lactate and cortisol biomarkers do not interfere with each other’s bioassays, which will eliminate false positives and ensure that the decrease in current seen is from the specific biomarker, by virtue of the well-known specificity of antibodies toward their counterpart antigens. This demonstrates that the Ab@e-RGO electrodes are highly selective toward the target molecules, cortisol, and lactate only.

The proposed simple electrochemical approach used in this study exhibited a quick-sensing response (≤ 1 min) without any sample pretreatment. The results obtained from our proposed electrochemical technique demonstrate its utility in the accurate determination of cortisol and lactate in spiked samples, being reproducible in the detection range, and the performance was validated by comparison with existing commercial ELISA techniques and also compared with published reports (tabulated in Table S1). The work for the Android OS-based mobile application is underway to communicate with the handheld potentiostat and display a final concentration. The development of the mobile app will enable the biosensor to be used as a POC device in remote areas.

## Conclusions

The present research work was carried out to explore the use of electroreduced 2D graphene sheets as a synergistic nanomaterial of choice for the fabrication of a dual electrochemical immunoassay system for cortisol and lactate determination. Being a 2D nanomaterial, e-RGO has amplified electronic properties and shows the synergistic effect of signal escalation as well as bioreceptor bioconjugation via a facile approach. Consequently, a high surface activity of e-RGO results in improved electrocatalytic characteristics, while the bioconjugation of proteins is facilitated by covalent chemistry approach. Thus, e-RGO was successfully synthesized by the electroreduction method and evaluated for potential as an immunoassay template. The e-RGO brings to the design of an immunoassay a simultaneous wide detection range capability to monitor cortisol and lactate quantitatively. The Ab@e-RGO immunoassay template also shows good selectivity and practical applicability, with the spiked sweat and saliva samples of cortisol and lactate. The graphene bionanotemplate can be an attractive candidate for POC diagnostic applications.

## Electronic supplementary material

Below is the link to the electronic supplementary material.

Supplementary material 1 (PDF 804 kb)

## References

[1] Levine A, Zagoory-Sharon O, Feldman R, Lewis JG, Weller A (2007). Measuring cortisol in human psychobiological studies. Physiol. Behav..

[2] Choi S, Kim S, Yang J-S, Lee J-H, Joo C, Jung H-I (2014). Real-time measurement of human salivary cortisol for the assessment of psychological stress using a smartphone. Sens. Bio-Sensing Res..

[3] Allen SE, Holm JL (2008). Lactate: physiology and clinical utility. J. Vet. Emerg. Crit. Care.

[4] Phypers B, Pierce JT (2006). Lactate physiology in health and disease. Contin. Educ. Anaesth.: Crit. Care Pain.

[5] Jeppesen JB, Mortensen C, Bendtsen F, Møller S (2013). Lactate metabolism in chronic liver disease. Scand. J. Clin. Lab. Invest..

[6] Rathee K, Dhull V, Dhull R, Singh S (2016). Biosensors based on electrochemical lactate detection: a comprehensive review. Biochem. Biophys. Rep..

[7] Sun B, Gou Y, Ma Y, Zheng X, Bai R, Ahmed Abdelmoaty AA, Hu F (2017). Investigate electrochemical immunosensor of cortisol based on gold nanoparticles/magnetic functionalized reduced graphene oxide. Biosens. Bioelectron..

[8] da Silva ETSG, Souto DEP, Barragan JTC, de F. Giarola J, de Moraes ACM, Kubota LT (2017). Electrochemical biosensors in point-of-care devices: recent advances and future trends. ChemElectroChem.

[9] Anastasova S, Crewther B, Bembnowicz P, Curto V, Ip HM, Rosa B, Zhong-Yang G (2017). A wearable multisensing patch for continuous sweat monitoring. Biosens. Bioelectron..

[10] Munje RD, Muthukumar S, Jagannath B, Prasad S (2017). A new paradigm in sweat based wearable diagnostics biosensors using room temperature ionic liquids (RTILs). Sci. Rep..

[11] Ajami S, Teimouri F (2015). Features and application of wearable biosensors in medical care. J. Res. Med. Sci..

[12] Wei J (2014). How wearables intersect with the cloud and the internet of things: considerations for the developers of wearables. IEEE Consum. Electron. Mag..

[13] Matzeu G, Florea L, Diamond D (2015). Advances in wearable chemical sensor design for monitoring biological fluids. Sens. Actuators B Chem..

[14] Parvaneh S, Grewal GS, Grewal E, Menzies RA, Talal TK, Armstrong DG, Sternberg E, Najafi B (2014). Stressing the dressing: assessing stress during wound care in real-time using wearable sensors. Wound Med..

[15] Khan MS, Misra SK, Wang Z, Daza E, Schwartz-Duval AS, Kus JM, Pan D, Pan D (2017). Paper-based analytical biosensor chip designed from graphene-nanoplatelet-amphiphilic-di-block-co-polymer composite for cortisol detection in human saliva. Anal. Chem..

[16] Kim KS, Lim SR, Kim SE, Lee JY, Chung CH, Choe WS, Yoo PJ (2017). Highly sensitive and selective electrochemical cortisol sensor using bifunctional protein interlayer-modified graphene electrodes. Sens. Actuators B Chem..

[17] Azzouzi S, Rotariu L, Benito AM, Maser WK, Ben Ali M, Bala C (2015). A novel amperometric biosensor based on gold nanoparticles anchored on reduced graphene oxide for sensitive detection of L-lactate tumor biomarker. Biosens. Bioelectron..

[18] Labroo P, Cui Y (2014). Graphene nano-ink biosensor arrays on a microfluidic paper for multiplexed detection of metabolites. Anal. Chim. Acta.

[19] Geim AK, Novoselov KS (2007). The rise of graphene. Nat. Mater..

[20] Yang Y, Asiri AM, Tang Z, Du D, Lin Y (2013). Graphene based materials for biomedical applications. Mater. Today.

[21] Tuteja SK, Kukkar M, Suri CR, Paul AK, Deep A (2015). One step in-situ synthesis of amine functionalized graphene for immunosensing of cardiac marker cTnI. Biosens. Bioelectron..

[22] Li S, Aphale AN, Macwan IG, Patra PK, Gonzalez WG, Miksovska J, Leblanc RM (2012). Graphene oxide as a quencher for fluorescent assay of amino acids, peptides, and proteins. ACS Appl. Mater. Interfaces.

[23] Tuteja SK, Sabherwal P, Deep A, Rastogi R, Paul AK, Suri CR (2014). Biofunctionalized rebar graphene (f-RG) for label-free detection of cardiac marker troponin I. ACS Appl. Mater. Interfaces.

[24] Pumera M (2011). Graphene in biosensing. Mater. Today.

[25] Wang G, Wang B, Park J, Wang Y, Sun B, Yao J (2009). Highly efficient and large-scale synthesis of graphene by electrolytic exfoliation. Carbon.

[26] Longo A, Verucchi R, Aversa L, Tatti R, Ambrosio A, Orabona E, Coscia U, Carotenuto G, Maddalena P (2017). Graphene oxide prepared by graphene nano-platelets and reduced by laser treatment. Nanotechnology.

[27] Tuteja SK, Duffield T, Neethirajan S (2017). Graphene-based multiplexed disposable electrochemical biosensor for rapid on-farm monitoring of NEFA and βHBA dairy biomarkers. J. Mater. Chem. B.

[28] Tuteja SK, Priyanka V, Bhalla A, Deep AK, Paul CR Suri (2014). Graphene-gated biochip for the detection of cardiac marker Troponin I. Anal. Chim. Acta.

[29] Ferrari AC, Robertson J (2001). Resonant Raman spectroscopy of disordered, amorphous, and diamond like carbon. Phys. Rev. B.

[30] Basirun WJ, Sookhakian M, Baradaran S, Mahmoudian MR, Ebadi M (2013). Solid-phase electrochemical reduction of graphene oxide films in alkaline solution. Nanoscale Res. Lett..

[31] Koinuma M, Ogata C, Kamei Y, Hatakeyama K, Tateishi H (2012). Photochemical engineering of graphene oxide nanosheets. J. Phys. Chem. C.

[32] Krishnamoorthy K, Veerapandian M, Yun K, Kim SJ (2013). The chemical and structural analysis of graphene oxide with different degrees of oxidation. Carbon.

