# Supplementary material for: Noninvasive Label-Free Detection of Cortisol and Lactate Using Graphene Embedded Screen-Printed Electrode
Source: Nanomicro Lett. 2018 Mar 2;10(3):41. doi: 10.1007/s40820-018-0193-5 (PMC6199085; doi:10.1007/s40820-018-0193-5)
Supplement: Supplementary file 1 — Supplementary material 1 (PDF 804 kb) [file 40820_2018_193_MOESM1_ESM.pdf]

Supporting Information for

# Non-Invasive Label-Free Detection of Cortisol and Lactate Using Graphene Embedded Screen-Printed Electrode

Satish K. Tuteja[1], Connor Ormsby[1], Suresh Neethirajan[1,*]
[1]BioNano Lab, School of Engineering, University of Guelph, ON, Canada (N1G 2W1)

*Corresponding author. E-mail: sneethir@uoguelph.ca
Tel: 1.519.824.4120

## Supplementary Figures and Table

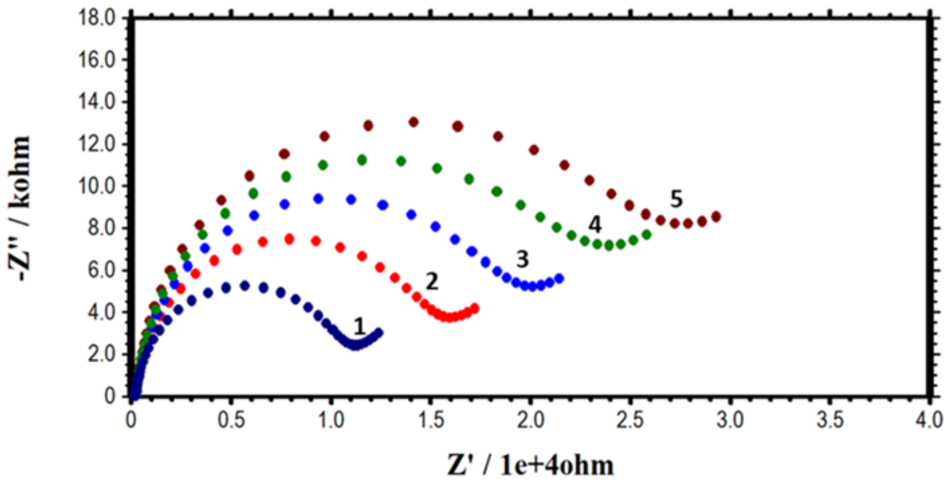


| Modified Electrode | $R_{ct}$ (diameter of semicircle) |
|---|---|
| 1. e-RGO | 9.52 |
| 2. Bare | 13.59 |
| 3. GO | 20.07 |
| 4. Ab@e-RGO | 23.4 |
| 5. Ag+Ab@e-RGO | 24.85 |

**Fig. S1** The step by step modification of SPE using electrochemical impedance spectroscopy (EIS)

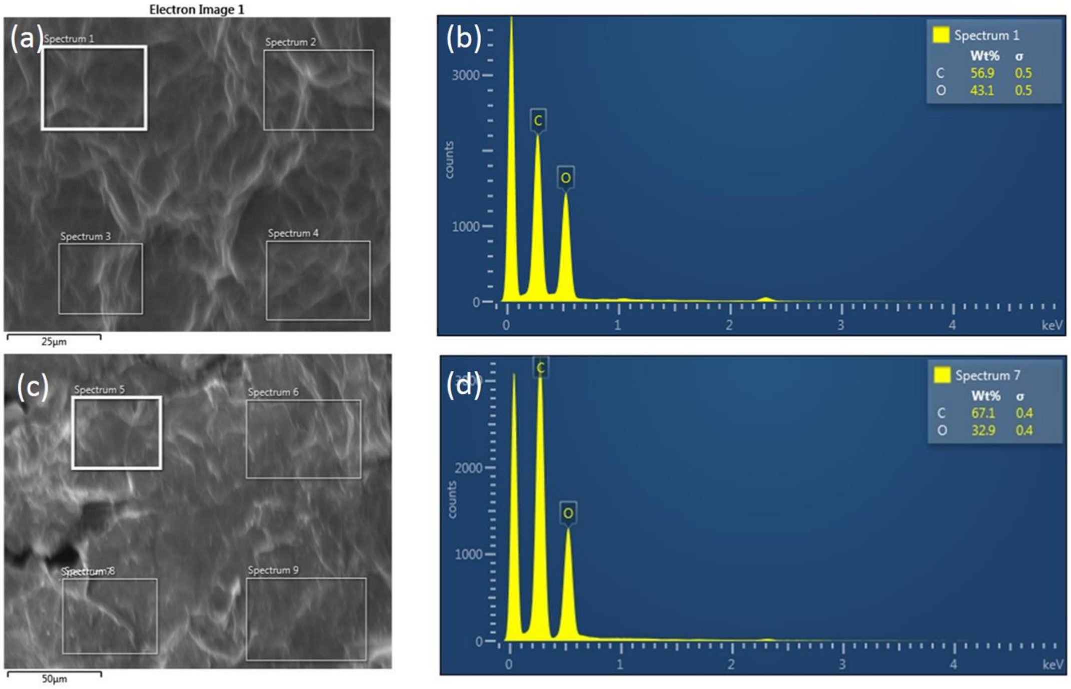


**Fig. S2 a, c** SEM microscopic images of GO and e-RGO. **b, d** EDS analyses of GO and e-RGO

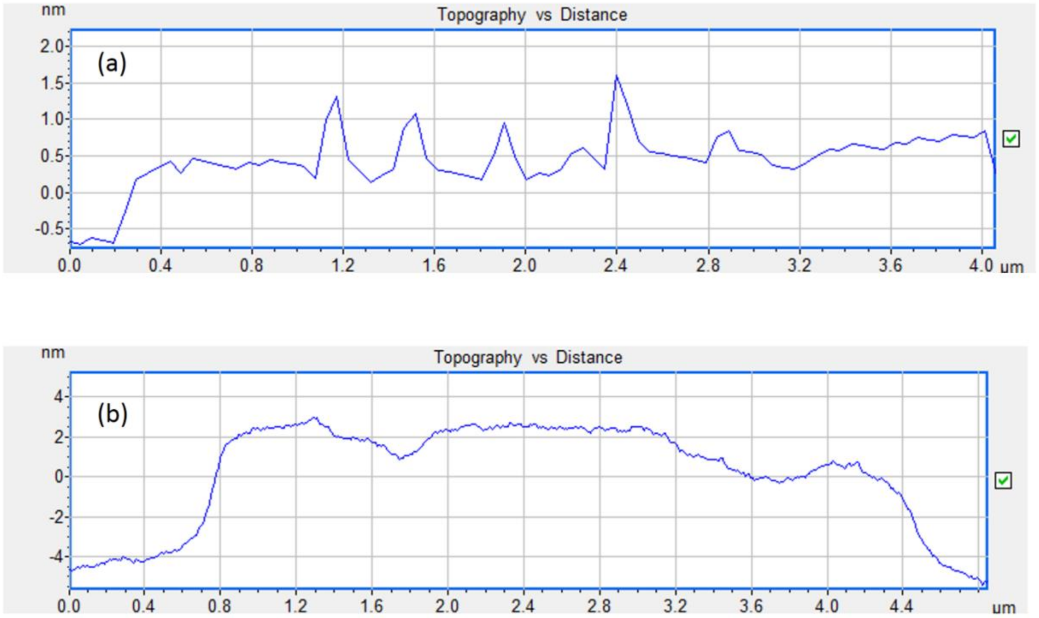


**Fig. S3 a, b** AFM line profile mapping of e-RGO deposited SPE surface

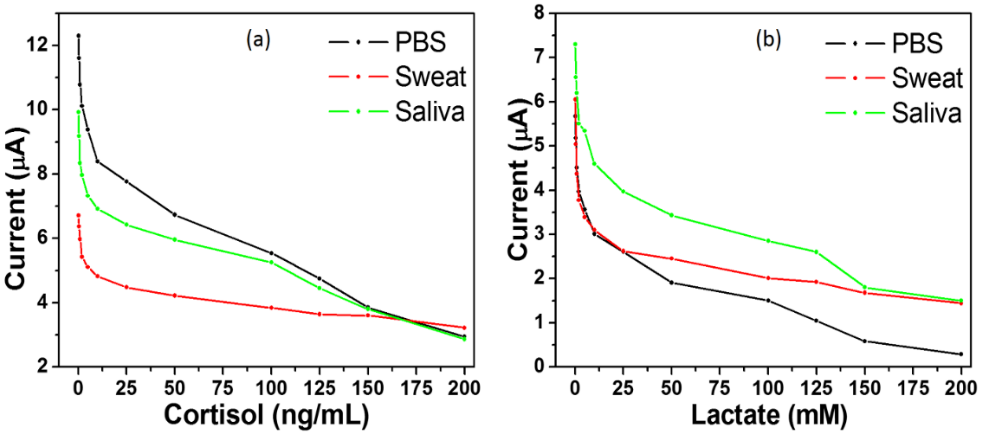


**Fig. S4 a** The calibration curves obtained from chronoamperometric responses of various cortisol antigen concentrations recorded using anti cortisol Ab@e-RGO SPE in standard PBS buffer, spiked sweat and saliva solution. **b** The calibration curves obtained from chronoamperometric responses of various lactate antigen concentrations recorded using anti lactate Ab@e-RGO SPE in standard PBS buffer, spiked sweat and saliva solution. The curves represent the decreasing relationship between current and antigen concentration generated by the insulating effect of the antigen-antibody complex.

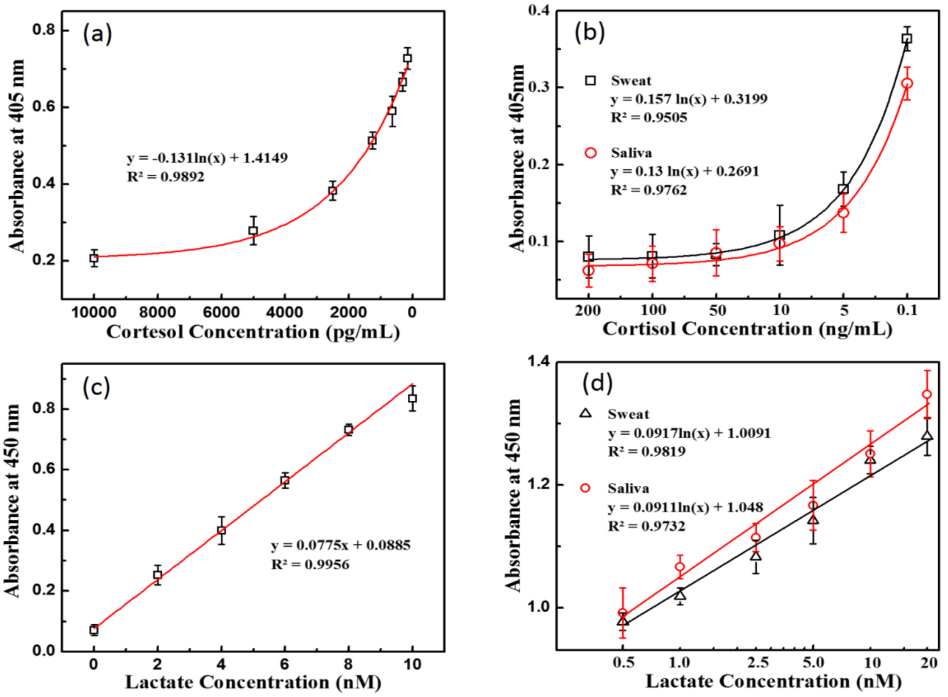


**Fig. S5 a** Analysis of standard samples with known concentrations of cortisol using color based absorbance (at 450 nm) with a cortisol commercial colorimetric kit. **b** Analysis of cortisol spiked sweat and saliva samples using cortisol commercial colorimetric kit. **c** Analysis of standard samples with known concentrations of lactate using color based absorbance (at 450 nm) with a lactate commercial colorimetric kit and **d** Analysis of lactate spiked sweat and saliva samples using lactate commercial colorimetric kit

**Table S1 Comparison with existing reports**

| Target | Platform | Technique | LOD | Ref. |
|---|---|---|---|---|
| Cortisol | SPR based anticortisol modified HC 80 on Au disk | Surface plasmon resonance | 4 µg $mL^{-1}$ | [1] |
| Cortisol | glucose oxidase (GOD)-cortisol conjugated labeled sensor | Calorimetric Chromatography | 1 ng $mL^{-1}$ | [2] |
| Cortisol | LFIA with anti-CAB | Chemiluminescence | 0.3 ng $mL^{-1}$ | [3] |
| Cortisol | 1D ZnO NRs and 2D ZnO NFs | Electrochemical | 0.36 mg $mL^{-1}$ | [4] |
| Lactate | Chitosan/MWCNTs with LOx | Electrochemical | 22.6 µM | [5] |
| Lactate | $TiO_2$-NPs, rGO with Lox | Electrochemical | 0.6 µM | [6] |
| Lactate | Prussian | Electrochemical | 0.01 mM | [7] |
| Lactate | HRP with LOx | Amperometric | 10 mmol $L^{-1}$ | [8] |
| Lactate | CNT-FET with LOx | Electrical | 1 pM | [9] |
| Cortisol and Lactate | e-RGO dual working area | Amperometric Detection | 0.1 ng $mL^{-1}$<br>1 mm | Present work |

## Validation with Commercial ELISA Kit

The proposed sensing assays were validated using the commercial lactate colorimetric assay kit II (Cat. no. K627-100, BioVision Incorporated, CA, USA) and cortisol ELISA kit (Cat. no. ADI-900-071, Enzo Life Sciences Inc., NY, USA). At first, different concentrated solutions of both lactate and cortisol were prepared using the sample diluent that was received with the commercial ELISA kits and the manufacturer's assay protocol was strictly maintained during the bioassay. Then, the same concentrated solutions of lactate and cortisol were prepared in complex media, i.e., sweat and saliva, and the assay protocol, as mentioned in the booklet, was strictly followed. The yellow color developed with different absorbance intensity related to the lactate and cortisol concentration, which was recorded at 450 nm in the 96-well plates. The sensitivity of the proposed electrochemical sensor was validated with the commercial colorimetric detection kit for both lactate and cortisol. The visual color response of the commercial kit was up to 0.1 ng $mL^{-1}$ and 0.5 nM for cortisol and lactate respectively, indicating that the prototype electrochemical method is sensitive and consistent with the commercial kits. The results are depicted in Fig. S5.

## References


[1] M. Frasconi, M. Mazzarino, F. Botrè, F. Mazzei, Surface plasmon resonance immunosensor for cortisol and cortisone determination. Anal. Bioanal. Chem. **394**(8), 2151-2159 (2009). https://doi.org/10.1007/s00216-009-2914-6

[2] M. Yamaguchi, S. Yoshikawa, Y. Tahara, D. Niwa, Y. Imai, V. Shetty, Point-of-use measurement of salivary cortisol levels. Sensors IEEE **14**, 343-346 (2009). https://doi.org/10.1109/ICSENS.2009.5398143

[3] M. Zangheri, L. Cevenini, L. Anfossi, C. Baggiani, P. Simoni, F. Di Nardo, A. Roda, A simple and compact smartphone accessory for quantitative chemiluminescence-based lateral flow immunoassay for salivary cortisol detection. Biosens. Bioelectron. **64**, 63-68 (2015). https://doi.org/10.1016/j.bios.2014.08.048

[4] P.K. Vabbina, A. Kaushik, N. Pokhrel, S. Bhansali, N. Pala, Electrochemical cortisol immunosensors based on sonochemically synthesized zinc oxide 1D nanorods and 2D nanoflakes. Biosens. Bioelectron. **63**(33), 124-130 (2015). https://doi.org/10.1016/j.bios.2014.07.026

[5] N. Hernández-Ibá-ez, L. García-Cruz, V. Montiel, C.W. Foster, C.E. Banks, J. Iniesta, Electrochemical lactate biosensor based upon chitosan/carbon nanotubes modified screen-printed graphite electrodes for the determination of lactate in embryonic cell cultures. Biosens. Bioelectron. **77**, 1168-1174 (2016). https://doi.org/10.1016/j.bios.2015.11.005

[6] E. Casero, C. Alonso, M.D. Petit-Domínguez, L. Vázquez, A.M. Parra-Alfambra, et al., Lactate biosensor based on a bionanocomposite composed of titanium oxide nanoparticles, photocatalytically reduced graphene, and lactate oxidase. Microchim. Acta **181**(1-2), 79-87 (2014). https://doi.org/10.1007/s00604-013-1070-z

[7] K. Petropoulos, S. Piermarini, S. Bernardini, G. Palleschi, D. Moscone, Development of a disposable biosensor for lactate monitoring in saliva. Sensors Actuators B Chem. **237**, 8-15 (2016). https://doi.org/10.1016/j.snb.2016.06.068

[8] F. Ghamouss, S. Ledru, N. Ruillé, F. Lantier, M. Boujtita, Bulk-modified modified screen-printing carbon electrodes with both lactate oxidase (LOD) and horseradish peroxide (HRP) for the determination of l-lactate in flow injection analysis mode. Anal. Chim. Acta **570**(2), 158-164 (2006). https://doi.org/10.1016/j.aca.2006.04.022

[9] S. Joshi, V.D. Bhatt, H. Wu, M. Becherer, P. Lugli, Flexible lactate and glucose sensors using electrolyte-gated carbon nanotube field effect transistor for non-invasive real-time monitoring. IEEE Sens. J. **17**, 4315 (2017). https://doi.org/10.1109/JSEN.2017.2707521
